# Arbuscular mycorrhizal fungi and production of secondary metabolites in medicinal plants

**DOI:** 10.1007/s00572-022-01079-0

**Published:** 2022-05-13

**Authors:** YanYan Zhao, Annalisa Cartabia, Ismahen Lalaymia, Stéphane Declerck

**Affiliations:** Université catholique de Louvain, Earth and Life Institute, Mycology, Croix du Sud 2, box L7.05.06, 1348 Louvain-la-Neuve, Belgium

**Keywords:** Arbuscular mycorrhiza fungi, Medicinal plants, Secondary metabolites, Hydroponics, Aeroponics, Hairy root cultures

## Abstract

Medicinal plants are an important source of therapeutic compounds used in the treatment of many diseases since ancient times. Interestingly, they form associations with numerous microorganisms developing as endophytes or symbionts in different parts of the plants. Within the soil, arbuscular mycorrhizal fungi (AMF) are the most prevalent symbiotic microorganisms forming associations with more than 70% of vascular plants. In the last decade, a number of studies have reported the positive effects of AMF on improving the production and accumulation of important active compounds in medicinal plants.

In this work, we reviewed the literature on the effects of AMF on the production of secondary metabolites in medicinal plants. The major findings are as follows: AMF impact the production of secondary metabolites either directly by increasing plant biomass or indirectly by stimulating secondary metabolite biosynthetic pathways. The magnitude of the impact differs depending on the plant genotype, the AMF strain, and the environmental context (e.g., light, time of harvesting). Different methods of cultivation are used for the production of secondary metabolites by medicinal plants (e.g., greenhouse, aeroponics, hydroponics, in vitro and hairy root cultures) which also are compatible with AMF. In conclusion, the inoculation of medicinal plants with AMF is a real avenue for increasing the quantity and quality of secondary metabolites of pharmacological, medical, and cosmetic interest.

## Introduction

Medicinal plants have been a valuable source of therapeutic agents to treat various ailments and diseases such as diarrhea, fever, colds, and malaria since ancient times (Dambisya and Tindimwebwa 2003; Ghiaee et al. 2014; Mathens and Bellanger 2010; Titanji et al. 2008). Nowadays, they also represent a source for the development of new drugs to cure important diseases such as cancer (Newman and Cragg 2007; Beik et al. 2020). Their therapeutic value often is attributed to the presence and richness of active compounds belonging to the secondary metabolism, such as alkaloids, flavonoids, terpenoids, and phenolics (Hussein and El-Anssary 2018). Today, up to 80% of people in developing countries are totally dependent on herbal drugs for their primary healthcare (Ekor 2014), and over 25% of prescribed medicines in developed countries have been derived from plants collected in the wild (Hamilton 2004).

Numerous methods, such as isolation from plants and other natural sources, synthetic chemistry, combinatorial chemistry, and molecular modeling, have been used for drug discovery (Ley and Baxendale 2002; Geysen et al. 2003; Lombardino and Lowe 2004). However, natural products, and particularly medicinal plants, remain an important source of new drugs, new drug leads, and new chemical entities (Newman et al. 2000, 2003; Butler 2004) because of their cultural acceptability, high compatibility, and adaptability with the human body compared to synthetic chemicals (Garg et al. 2021). According to the International Union for Conservation of Nature and the World Wildlife Fund (Chen et al. 2016), an estimate of as many as 80,000 flowering plant species are used for medicinal purposes. For several thousands of plants worldwide, the activity or composition in bioactive compounds remains poorly documented, requiring further in-depth analysis to fully exploit their medicinal potential (Ali 2019).

In nature, plants are associated with an overwhelming number of beneficial microorganisms (e.g., endophytic or symbiotic bacteria and fungi) that play a significant role in plant health, development, and productivity, and in the modulation of metabolite synthesis (Berendsen et al. 2012; Panke-Buisse et al. 2015; Mendes et al. 2011; Castrillo et al. 2017; de Vries et al. 2020; Brader et al. 2014; Compant et al. 2021). Among these are the arbuscular mycorrhizal fungi (AMF), a ubiquitous group of soil microorganisms, forming symbiosis with more than 70% of vascular plants (Brundrett and Tedersoo 2018). Arbuscular mycorrhizas are characterized by the formation of finely branched structures called arbuscules within root cortical cells of host plants (Coleman et al. 2004), which are the site of bidirectional transport, i.e., minerals from the fungal cell to the plant cell and carbon compounds in the opposite direction.

The establishment of the AMF symbiosis requires recognition between the two partners. Lipochitooligosaccharides, the so-called Myc factors, are perceived by the plant in response to signaling molecules (i.e., strigolactones) released by the roots (Akiyama and Hayashi 2006). After reciprocal recognition, AMF hyphae form a hyphopodium on the root epidermis and colonize the root cortex. At the same time, fungal hyphae spread into the surrounding soil as an extensive extraradical mycelium, representing 9 to 55% of the total soil microbial biomass (Olsson et al. 1999). This dense extraradical mycelium considerably enhances the access of roots to water and mineral nutrients (e.g., P, N, K, Ca, S, Zn, Cu), often increasing plant biomass (Smith and Read 2008; Bowles et al. 2016) and quality of crops (Baum et al. 2015; Bona et al. 2016; Noceto et al. 2021). Moreover, this extraradical mycelium modifies the soil structure (Chen et al. 2018), which improves soil quality and fertility (Zou et al. 2016; Thirkell et al. 2017). AMF also are well known to improve plant resistance or tolerance to stress conditions, such as drought, salinity, nutrient deprivation, extreme temperatures, heavy metals, pests, and diseases (Ahanger et al. 2014; Salam et al. 2017; Porcel et al. 2012; Cicatelli et al. 2014). In addition to these benefits, they also quantitatively and qualitatively could affect the production of secondary metabolites produced by their hosts (Ahanger et al. 2014; Salam et al. 2017; Porcel et al. 2012; Cicatelli et al. 2014; Kaur and Suseela 2020).

Taber and Trappe (1982) were the first to document the presence of AMF in a medicinal plant (in their study conducted on ginger growing in the Fiji Islands and Hawaii). Since then, most medicinal plants were found capable of associating with mycorrhizal fungi (Chen et al. 2014). Recently, single or combinations of AMF have been inoculated to various medicinal plants to investigate their impact on plant biomass as well as on phytochemical constituents in seeds, fruits, leaves, shoots, and roots (e.g., Rydlová et al. 2016; Kapoor et al. 2004; Selvaraj et al. 2009; Dave et al. 2011; Zubek et al. 2012). The majority of studies revealed that AMF were able to enhance plant biomass as well as to promote the accumulation of several active compounds. For example, Lazzara et al. (2017) reported an increased above- and belowground biomass in *Hypericum perforatum* associated with a mixture of nine different AMF species. Interestingly, the concentrations of pseudohypericin and hypericin, two anthraquinone derivatives that exhibit important photodynamic, antiviral, antiretroviral, antibacterial, antipsoriatic, antidepressant, and antitumoral biological activities (Zubek et al. 2012; Bombardelli and Morazzoni 1995; Gadzovska et al. 2005; Guedes and Eriksson 2005), were increased by 166.8 and 279.2% in the AMF-colonized plants as compared to non-mycorrhizal controls (Lazzara et al. 2017). However, these results should not obviate other studies in which no effects on biomass were reported. For instance, Nell et al. (2010) found that AMF colonization decreased the biomass of rhizomes and roots of *Valeriana officinalis*, while significantly increasing the levels of sesquiterpenic acids. Another study by Engel et al. (2016) reported an increased content of rosmarinic acid and lithospermic acid A isomer (two phenolic compounds) in *Melissa officinalis*, while both compounds were diminished in *Majorana hortensis*, in the presence of three mixtures of AMF. More recently, Duc et al. (2021) showed that a mixture of different AMF species improved the salt stress tolerance of *Eclipta prostrata*, inducing major changes in polyphenol profile.

In this publication, we provide a thorough review of the literature on AMF mediation of secondary metabolites production in medicinal plants. We also review the different methods that are used to increase/stabilize the production of secondary metabolites. Indeed, the quantity and quality of secondary metabolites obtained from plants grown in natural habitats are critically influenced by various abiotic and biotic stresses (e.g., drought, extreme temperatures, and pathogen attack). This results in high variability of bioactive substances and influences the metabolic pathways responsible for the accumulation of the related natural compounds (Dayani and Sabzalian 2017; Giurgiu et al. 2017; Ramakrishna and Ravishankar 2011). Therefore, we additionally review the most widely used methods of cultivation (i.e., greenhouse, hydroponics, aeroponics, in vitro and hairy root cultures (HRCs)) of medicinal plants, and we investigate their possible application to AMF to further increase the quantity and quality of secondary metabolites produced.

## Effect of AMF on growth and secondary metabolite production of medicinal plants

Since the pioneer work of Wei and Wang (1989, 1991), reporting the positive effect of AMF inoculation of *Datura stramonium* and *Schizonepeta tenuifolia* on the production of active compounds, numerous studies have been conducted. The literature focusing on AMF in medicinal plants involves 81 plant species belonging to 28 families (Table 1). These medicinal plants present different characteristics to be studied with AMF: important medicinal herbs to treat certain disease such as *Artemisia annua* producing artemisin to treat malaria in developing countries (Domokos et al. 2018); important condiment plants such as *Allium sativum* in India (Borde et al. 2009); rare plant species difficult to culture such as *Arnica montana* (Jurkiewicz et al. 2011); aromatic plants to produce essential oil widely used in the pharmaceutical, cosmetic, and food industries such as most plant species from the Apiaceae (*Anethum graveolens* and *Coriandrum sativum* (Rydlová et al. 2016)) and the Lamiaceae (*Origanum vulgare* (Karagiannidis et al. 2012)); and health foods such as *Dioscorea* spp. yam (Lu et al. 2015). For the majority of these plants, studies were focused on the effects of AMF on biomass increase and production of bioactive compounds simultaneously (45 studies) or only focused on bioactive compound production (30 studies). For a few other studies, the attention was focused on AMF community composition (four studies) or on the effects of AMF on plant growth under different conditions (15 studies) (Table 1). Among the AMF species tested, *Funneliformis mosseae*^1^ is the most investigated one (25 studies), followed by *Rhizophagus intraradices* (16), *Claroideoglomus etunicatum* (14), *Rhizophagus fasciculatus* (14), *Rhizophagus irregularis* and *Rhizophagus clarus* (six studies each), and *Gigaspora margarita* (five studies) (Table 1). Only a few medicinal plant species were inoculated with AMF present in the soil native to those plants, while the vast majority were inoculated with commercial inoculants.

**Table 1:** Detailed summary of studies on the relationship between AMF and medicinal plants

**Plant family**	**Plant species**	**AMF**^**a**^	**Secondary metabolites group and active ingredients**	**Medicinal value of the plant**	**Plant organ studied**	**Change in secondary metabolite production**	**Change in plant growth/biomass**	**Reference**
Alliaceae	*Allium sativum*	*Rhizophagus fasciculatus*	Alliin	Antibacterial, antifungal, antiviral, antiprotozoal, antioxidative, and anticancerogenic properties; against arteriosclerosis and high blood pressure	Leaves, flowers, and cloves	Significant increase	Increase in plant height, total biomass and bulb diameters, bulb weight, and yield	Borde et al. (2009)
Amaranthaceae	*Achyranthes aspera*	*Rhizophagus fasciculatus*	Flavonoids	Treatment of cough, bronchitis, rheumatism, malarial fever, dysentery, asthma, hypertension and diabetes (Bhosale et al. 2012)	______^b^	Increased the contents of active principles	Positive effect on plant growth parameters	Tejavathi and Jayashree (2011)
Anacardiaceae	*Myracrodruon* *urundeuva*	*Acaulospora longula*	Total phenols and flavonoids	Anti-inflammatory, anti-ulcer, astringent, anti-allergic, and antidiarrheal activities (Teixeira et al. 2020)	Leaves	81.03% increased	Promote plant growth	Oliveira et al. (2013)
Apiaceae	*Angelica* *dahurica*	*Glomus* spp.	Imperatorin and total coumarins	Treatment for colds, headache, dizziness, toothache, supraorbital pain, nasal congestion, acne, ulcer, carbuncle, and rheumatism (Lee et al. 2015)	Root, seed, and fruit	Significant increase	Plant growth and biomass promoted	Zhao and He (2011)
	*Angelica archangelica*	*Funneliformis mosseae*	Monoterpenoid and coumarin	Dyspeptic complaints such as mild gastrointestinal spasms, sluggish digestion, flatulence and feeling of fullness, loss of appetite, anorexia and bronchitis	Rhizome and roots	A marked increase in yield	Biomass increased	Zitterl-Eglseer et al. (2015)
	*Anethum graveolens*	*Glomus macrocarpum*, *Rhizophagus fasciculatus*	Anethole	Treatment for abdominal discomfort and colic and also for promoting digestion (Jana and Shekhawat 2010)	Seed	90% increased	Improved the growth	Kapoor et al. (2002)
	*Bupleuruin scorzonerifolium*	______	Flavonoids	Treating cold, fever, chest pain, irregular menstruation, uterine fall off and rectocele	______	______	______	Teng and He (2005)
	*Coriandrum* *sativum*	*Glomus hoi*	β-Caryophyllene, p-cymene, geraniol	Antioxidant, antidiabetic, hepatoprotective, antibacterial, and antifungal activities (Asgarpanah and Kazemivash 2012)	Seeds or leaves	Significant improvement	______	Rydlová et al. (2016)
	*Foeniculum vulgare*	*Glomus macrocarpum* and *Rhizophagus fasciculatus*	Essential oil concentration	Used for digestive, endocrine, reproductive, and respiratory systems (Badgujar et al. 2014)	Seeds	Significantly increased	Improved plant growth	Kapoor et al. (2004)
	*Trachyspermum ammi*	*Rhizophagus fasciculatus*	Thymol	Antifungal, antioxidant, antimicrobial properties and used for antinociceptive, hypolipidemic, antihypertensive, antispasmodic, broncho-dilating actions, antilithiasis, and diuretic (Bairwa et al. 2012)	Fruits	72% increased	______	Kapoor et al. (2002)
Apocynaceae	*Catharanthus roseus*	*Glomus spp.*	Vinblastine and vincristine, rutin, quercetin, and kaempferol	Treatment of diuretic, hemorrhagic, wound healing, coughs, sore throats, lung infections, and diabetes (Gupta et al. 2017)	Aerial part	Significant increase	______	Andrade et al. (2013)
	*Gymnema sylvestre*	*Rhizophagus fascuculatus* and *Funneliformis mosseae*	Gymnemic acid	Control diabetes mellitus	Shoots and leaves	Positive increased	Higher shoot and root length and fresh and dry weight	Zimare et al. (2013)
Araliaceae	*Panax ginseng*	______	______	Reinforcing vital energy and restoring physiological weakness and possess antioxidation, anti-inflammatory, antiallergic, antidiabetic, and anticancer properties (Kim et al. 2018)	______	______	Plant seedlings biomass Significantly increased	Cho et al. (2009)
		*Rhizophagus intraradices*	Ginsenosides		Roots	Increased total content	______	Tian et al. (2019)
	*Panax notoginseng*	______	______	Used to staunch bleeding, and invigorating and supplementing blood (Yang et al. 2014)	______	______	Only AMF community study from plant	Ren et al. (2007)
Araceae	*Pinellia ternate*	*Rhizophagus intraradices*, *Funneliformis mosseae*	l-Ephedrine and guanosine	Treating cough and vomiting	Tubers	Significant increase	Increasing fresh weight and dry weight	Guo et al. (2010)
	*Acorus calamus*	*Funneliformis mosseae* and *Acaulospora laevis*	______	Anti-spasmodic and anti-anthelmintic properties and also used for treatment of epilepsy, mental ailments, chronic diarrhea, dysentery, bronchial catarrh, intermittent fevers, and tumors	______	______	Significant increase in plant height, plant spread, number of leaves per plant, and leaf area	Yadav et al. (2011)
Asteraceae	*Atractylodes macrocephala*	*Funneliformis mosseae*	Atractylol	Strengthening the spleen, benefiting vital energy, eliminating dampness, hidroschesis, and soothing fetuses (Gu et al. 2019)	Rhizome	Significant increase	______	Lu and He (2005)
	*Atractylodes lancea*	*Funneliformis mosseae*	______	Used to treat rheumatic diseases, digestive disorders, night blindness, and influenza and also exert anti-cancer, anti-obesity, and anti-inflammatory effects (Jun et al. 2018)	______	No effect on essential oil contents	Improved plant growth	Guo et al. (2006)
		*Claroideoglomus etunicatum*, *Glomus tortuosum*, and *Funneliformis mosseae*	Essential oils, hinesol, β-eudesmol, and atractylodin		______	Increased	Increased the survival rate of seedlings, plant height, root length, and leaf number significantly increased	Liang et al. (2018)
	*Artemisia annua*	*Rhizophagus irregularis*	Artemisinin content	Treat fever, inflammation, malaria, cough, stomach and intestinal upset	Leaves	17% increased	Significant increase in fresh and dry plant biomass	Domokos et al. (2018)
	*Arnica montana*	*Funneliformis geosporum*, *Funneliformis constrictum*	Sesquiterpene lactones	Stimulate blood flow, promote healing, and soothe arthritic pains	Fresh or dried flower	Significant increase	______	Jurkiewicz et al. (2011)
		several Glomus strains	Phenolic acids		Roots	Increased concentration	______	Jurkiewicz et al. (2011)
	*Artemisia umbelliformis*	*Planticonsortium tenue*, *Rhizophagus intraradices*, *Claroideoglomus claroideum/etunicatum*, and a new *Acaulospora species*	Essential oil E-β-ocimene	Against coughs	Shoots	Significantly increased	Increase of P concentration in shoots	Binet et al. (2011)
	*Baccharis trimera*	*Rhizophagus clarus*	Phenolics	Antioxidant, anti-microbial, anti-fungal, anti-parasitic and anti-inflammatory properties, and used for gastric and hepatic-protector (Rabelo and Costa 2018)	______	Marked increases	Dry weight of the aerial part and height of plants increased	Freitas et al. (2004)
	*Cynara cardunculus*	*Rhizophagus intraradices* and *Funneliformis mosseae*	Phenolics	Prevent carcinogenesis and atherosclerosis	Leaves and flowers	Marked increases	______	Ceccarelli et al. (2010)
			Total phenolic content		______	No impact	Significantly increased plant yield	Colonna et al. (2016)
	*Echinacea purpurea*	*Rhizophagus* *intraradices*	Phenolics and cichoric acid	Treatment of toothache, bowel pain, snake bite, skin disorders, seizure, chronic arthritis, and cancer (Grimm and Muller 1999)	Root and aerial parts	Significant increase	Plant growth increased	Araim et al. (2009)
	*Eclipta alba*	*Glomus aggregatum*, *Funneliformis mosseae*, and *Rhizophagus fasciculatus*	Flavonoids	Treatment of gastrointestinal disorders, respiratory tract disorders (including asthma), fever, hair loss and graying of hair, liver disorders (including jaundice), skin disorders, spleen enlargement, and cuts and wounds (Jahan et al. 2014)	______	Increased	Positive effect on plant growth	Tejavathi and Jayashree (2011)
	*Eclipta prostrata*	*Rhizophagus irregularis*, *Funneliformis mosseae*, *Claroideoglomus etunicatum*, *Claroideoglomus claroideum*, *Rhizoglomus microaggregatum*, and *Funneliformis geosporum*	Scopolamine	treatment of diabetes type II, dizziness, hemoptysis, and liver diseases	Leaves	0.34% increased	______	Vo et al. (2019)
			Quercetin		Whole plant	0.87% increased	______	Vo et al. (2019)
	*Inula ensifolia*	*Rhizophagus clarus*	Thymol derivatives	Possess antiproliferative activity against human cancer	Roots	Increased	______	Zubek et al. (2010)
	*Stevia rebaudiana*	*Rhizophagus fasciculatus*	Stevioside, rebaudioside-A	Used as a substance strengthening the heart, the circulatory system, and regulating blood pressure (Marcinek and Krejpcio 2016)	Leaves	Significant increase	______	Mandal et al. (2013)
		*Rhizophagus irregularis*	______		______	Positive increase	Leaf dry biomass increased	Tavarini et al. (2018)
	*Spilanthes acmella*	*Funneliformis mosseae* and *Acaulospora laevis*	______	Antiseptic, antibacterial, antifungal, and antimalarial properties and used as remedy for toothache, flu, cough, rabies diseases, and tuberculosis	______	______	Improved the survival rate, plant growth, and biomass yield of micropropagated plantlets	Yadav et al. (2012)
	*Tagetes erecta*	______	______	Used as antiseptic and in kidney troubles, muscular pain, and piles, and applied to boils and carbuncles (Singh et al. 2020)	______	______	Positively improved plant growth, and flower quality under drought stress	Asrar and Elhindi (2010)
	*Wedilia chinensis*	*Rhizophagus fasciculatus*	Total phenols, ortho dihydroxy phenols, flavonoids, alkaloids, tannins, and saponins	Treatment of bites, stings, fever, infection, kidney dysfunction, cold, wounds, and amenorrhea problems (Rehana and Nagarajan 2018)	Seedlings	Increased	______	Nisha and Kumar (2010)
Burseraceae	*Commiphora leptophloeos*	*Gigaspora albida* and *Claroideoglomus etunicatum* (native)	Total phenols and tannins	Treatment of bronchitis, cough, renal problems, general inflammation, and stomachache	Seedling, leaves	Significant increased	______	Lima et al. (2017)
Caprifoliaceae	*Valeriana jatamansi*	*Rhizophagus* *intraradices*	Gallic acid, chlorogenic acid, catechin, hydroxyl benzoic acid	Possess sedative, neurotoxic, cytotoxic, antidepressant, antioxidant, and antimicrobial activities (Jugran et al. 2019)	Rhizome and root	Significant increase	Significant increase in aboveground fresh and dry weight, and belowground fresh and dry weight	Jugran et al. (2015)
	*Valeriana officinalis*	*Rhizophagus intraradices*	Valerenic acid	Possess sedative and antispasmodic and sleep-inducing effects (Mungali and Tripathi 2021)	Roots	Relative increasing	Biomass of rhizomes and roots negatively effected	Nell et al. (2010)
Colchicaceae	*Gloriosa superba*	*Funneliformis mossae*, *Rhizophagus fasciculatus*, *Gigaspora margarita*, and *Gigaspora gilmorei*	Colchicine content	Treatment of gout, rheumatic arthritis, diseases of the skin and liver	Tubers	Increased	Improved plant growth	Pandey et al. (2014)
Dioscoreaceae	*Dioscorea* spp. yam	*Rhizophagus clarus*, *Claroideoglomus etunicatum*, *Rhizophagus fasciculatus*, *Gigaspora* sp., *Funneliformis mosseae*, and *Acaulospora* sp.	Polyphenols, flavonoids, and anthocyanin	Anti-oxidative property to inhibit lipid peroxidation, resist the attack of free radicals, diminish low-density lipoproteins (LDLs), and reduce the occurrence of cardiovascular diseases	Bulbils	Significantly increased	Tube weights significantly increased	Lu et al. (2015)
Euphorbiaceae	*Euphorbia hirta*	*Funneliformis mosseae*	Phenols, flavonoids, alkaloids, and terpenoids	Treatment for respiratory ailments (cough, coryza, bronchitis, and asthma), worm infestations in children, dysentery, jaundice, pimples, gonorrhea, digestive problems, and tumors (Kumar et al. 2010)	______	Increased	Positive effect on plant growth parameters	Tejavathi and Jayashree (2011)
Fabaceae	*Astragalus membranaceus*	______	______	Increasing telomerase activity and posing antioxidant, anti-inflammatory, immunoregulatory, anticancer, hypolipidemic, antihyperglycemic, hepatoprotective, expectorant, and diuretic effects (Liu et al. 2017)	______	______	AMF community study	Liu and He (2008)
	*Anadenanthera* *colubrina*	*Acaulospora longula* and *Gigaspora albida*	Catechin	Treatment for respiratory problems and inflammations (Monteiro et al. 2006)	Bark and leaves	Significant increase	Proteins and carbohydrates were significantly increased	Pedone- Bonfim et al. (2013)
	*Castanospermum austral*	*Rhizophagus intraradices* and *Gigaspora margarita*	Castanospermine	Possess anti-cancer and anti-inflammatory properties and as HIV inhibitors and treatment of AIDS	Seeds	Significant increase with *R. intraradices*	Increased the growth and P contents	Abu-Zeyad et al. (1999)
	*Glycyrrhiza inflata*	______	______	Clearing away toxic materials, eliminating phlegm, and relieving cough	______	______	Study under water stress	Liu and He (2009)
	*Glycine max*	*Funneliformis mosseas*	Isoflavonoids	Reduction of different types of cancer, cardiovascular diseases, postmenopausal problems, diabetes, and some neurodegenerative disorders (Ahmad et al. 2014)	Roots, seeds, leaves, and flowers	Significant increase	______	Morandi and Bailey (1984)
	*Glycyrrhiza glabra*	*Glomus hoi*, *Claroideoglomus etunicatum*, *Claroideoglomusclaroideum*, *Rhizophagus irregularis*, and *Acaulospora delicata*	Glycyrrhizic acid	Antiviral effects and act as a multifunctional drug carrier	Roots	Increased	______	Johny et al. (2021)
	*Glycyrrhiza uralensis*	*Funneliformis mosseae*	Contents of glycyrrhizic acid, liquiritin, isoliquiritin, and isoliquiritigen	Having immune-modulating and anti-tumor potential (Ayeka et al. 2016)	Roots	Significantly enhanced	Significantly increased the shoot and root biomass	Chen et al. (2017)
	*Libidibia ferrea*	*Claroideoglomus etunicatum*	Total flavonoids	Posing antiulcerogenic, antiinflammatory, anti-cancerogenic, anti-histaminic, antimicrobial, anti-coagulant, and cicatrizing properties	Leaves	Increased	Improving the production of seedlings, a larger stem diameter, higher chlorophyll a leaf content	Silvia et al. (2014)
		*Claroideoglomus etunicatum* and *Acaulospora* *longula*	Flavonoids		Stems, bark, and leaves	Significantly increased	______	Dos Santos et al. (2017)
		*Acaulospora longula*	Tannins		______	Significantly increased	______	Dos Santos et al. (2017)
	*Medicago sativa*	*Rhizophagus* *intraradics*	Formononetin	Antioxidant, anti-inflammatory, immunomodulatory, and anticancer properties (Zagórska-Dziok et al. 2020)	Roots	Significant increase	______	Volpin et al. (1994)
	*Prosopis* *laevigata*	*Gigaspora rosea*	Trigonelline	Cardioprotection potential and treatment of heart diseases, throat infections, dysentery, and eye inflammations (Matta et al. 2017)	Roots and leaves	1.8-fold increase in roots	______	Rojas-Andrade et al. (2003)
Ginkgoaceae	*Ginkgo biloba*	*Funneliformis mosseae*, *Rhizophagus intraradices*, and *Diversispora epigaea*	______	Regulating cerebral blood flow, protection against free radicals, and delaying the progress of dementia and diabetes (Isah 2015)	______	______	Plant seedling growth significantly increased	Qi et al. (2002, 2003)
Hypericaceae	*Hypericum* *perforatum*	*Rhizophagus intraradices* alone or mixture of *Funneliformis constrictum*, *Funneliformis geosporum*, *Funneliformis mosseae*, and *Rhizophagus intraradices*	Naphthodianthrone-es, hypericin, and pseudohypericin	Possess sedative and astringent properties and utilized for excitability, neuralgia, anxiety, and depression	Shoots	Higher concentration	No impact on shoot biomass	Zubek et al. (2012)
Hypoxidaceae	*Curculigo orchioides*	Crude consortium of AMF spores isolated from rhizosphere soil of *C. orchioides*	______	Anticancerous properties	______	______	Increase biomass production, number of leaves and roots per plant, and higher concentrations of photosynthetic pigments as well as minerals	Sharma et al. (2008)
Lamiaceae	*Coleus forskohlii*	*Glomus bagyarajii* and *Scutellospora calospora*	Forskolin	Treatment of eczema, asthma, psoriasis, cardiovascular disorders, and hypertension (Kavitha et al. 2010)	Roots	Increased	Positive effect on plant growth	Sailo and Bagyaraj (2005)
	*Leucas aspera*	*Funneliformis mosseae*	Alkaloids	Carminative, antihistaminic, antipyretic, and antiseptic properties to treat jaundice, anorexia, dyspepsia, fever, helminthic manifestation, respiratory and skin diseases (Nirmala and Kanchana 2018)	______	Increased	Enhanced growth and total biomass	Tejavathi and Jayashree (2011)
	*Mentha arvensis*	*Rhizophagus fasciculatus*	Terpenes content	Used for stomach problems, allergy, liver and spleen disease, asthma, and jaundice (Thawkar et al. 2016)	Aerial parts	Significantly increased	Significantly increasing plant height, fresh herbage and dry matter yield	Gupta et al. (2002)
	*Mentha spicata*	Commercial AMF consortium “Rhizagold”	______	Antiseptic, restorative, carminative, and antispasmodic properties	______	______	Significantly positive effect of increasing various plant growth parameters	Birje and Golatkar (2016)
	*Melissa* *officinalis*	*Claroideoglomus etunicatum*, *Claoideoglomus claroideum*, and *Rhizophagus intraradices*	Citronellal and neral	To treat nervous disturbances (anxiety, insomnia, and stress) and gastrointestinal disorders and possess sedative, spasmolytic, antimicrobial, antioxidant, and antitumoral actions	Leaves	Increased	No impact	Engel et al. (2016)
	*Ocimum* *basilicum*	*Gigarpora margarita* and *Gigaspora rosea*	Linalool and geraniol	Treatment for headaches, coughs, diarrhea, constipation, warts, worms, and kidney malfunctions (Joshi 2014)	Seeds	Significant increase	Plant growth parameters and yield increased	Rasouli- Sadaghiani et al. (2010)
		*Funneliformis caledonium*	Rosmarinic and caffeic acids		Shoots	Increased	______	Toussaint et al. (2007)
	*Origanum onites*	*Claroideoglomus etunicatum*	Total essential oil production	Treatment of indigestion, coughs, and toothache, and to stimulate menstruation	Leaves	Increased	Significantly higher shoot and root dry weight	Karagiannidis et al. (2012)
	*Origanum vulgare*	*Claroideoglomus etunicatum*	Essential oil composition of p-cymene, and γ-terpinene	Treatment for indigestion, coughs, and toothache, and to stimulate menstruation	Leaves	Increased	Significantly higher shoot and root dry weight	Karagiannidis et al. (2012)
	*Plectranthus amboinicus*	*Rhizophagus clarus*	Carvacrol, trans-caryophyllene, α-Bergamotene and α-humulene	Possess digestive, expectorant, antispasmodic, healing, and antiseptic actions	Shoots	Significant improvement	Improved shoot dry matter, root dry matter and total dry matter	Merlin et al. (2020)
	*Pogostemon cablin*	*Claroideoglomus etunicatum*	Essential oils	Used to treat nausea, diarrhea, colds, and headaches	______	Increased essential oil content	Greater plant height, number of branches and spread, biomass	Arpana et al. (2008)
		*Acaulospora laevis*, *Funneliformis mosseae*, and *Scutellospora calaspora*	Patchoulol		Leaves	Significant Improvement	______	Singh et al. (2012)
	*Satureja macrostema*	*Rhizophagus irregularis*	β-Linalool, menthone, pulegone, and verbenol acetate	Antimicrobials	Aerial parts	Significantly increased	Significantly increased biomass, shoot and root length	Carreón-Abud et al. (2015)
	*Salvia officinalis*	*Rhizophagus clarus*	Essential oil camphor, α-humulene, viridiflorol, manool, α-thujone, and β-thujone	Treatment of different kinds of disorders including seizure, ulcers, gout, rheumatism, inflammation, dizziness, tremor, paralysis, diarrhea, and hyperglycemia (Ghorbani and Esmaeilizadeh 2017)	Shoots	Increased	Plant biomass increased	Sete da Cruz et al. (2019)
	*Salvia miltiorrhiza*	*Funneliformis geosporum* or *Acaulospora laevis*	Total phenolic acids	Treatment of menstrual disorders, cardiovascular, and cerebrovascular disease	Roots	Significant increase	Roots biomass, fresh and dry weight of the plant effectively increased	Wu et al. (2021)
	*Scutelleria integrifolia*	______	______	A strong emmenagogue and as a female medicinal herb	______	______	Positive effects on micropropagated plantlet growth, particularly root development	Joshee et al. (2007)
	*Schizonepeta tenuifolia*	______	Essential oil	Used for headaches, colds, allergies, and eczema (Jeon et al. 2019)	______	Increased	______	Wei and Wang (1991)
	*Thymus daenensis*	*Funneliformis mosseae* and *Rhizophagus intraradices*	Essential oils	Possess digestive, carminative, antitussive, antispasmodic, and expectorant attributes (Elahian et al. 2021)	______	Improve essential oil under drought stress	______	Arpanahi et al. (2020)
	*Thymus vulgaris*	*Funneliformis mosseae*	Thymol, p-cymene, and γ-terpinene	Possess antiseptic, antibacterial, antifungal, antispasmodic, antitussive, expectorant, and analgesic properties		Increased	Improved yield under drought condition	Machiani et al. (2021)
Leguminosae	*Puerraria lobata*	______	______	To relieve body heat, eye soring, dry mouth, headache associated with high blood pressure, and stiff neck problems (Liu et al. 2019)	______	______	AMF community study	Wang et al. (2006)
Oleaceae	*Forsythia suspense*	*Rhizophagus fasciculatus* and *Funneliformis constrictum*	______	Anti-inflammatory, antioxidant, antibacterial, anti-cancer, anti-virus, anti-allergy, and neuroprotective effects (Wang et al. 2018)	______	______	Strengthen the anti-drought of the seeding	Zhao et al. (2007)
Poaceae	*Cymbopogon citratus*	*Funneliformis mosseae*	Essential oils Geranial, neral, and β-pinene	To treat cough, cold, rheumatism, digestive problems, bladder issues, toothache, and swollen gums	Aerial Parts	Enhanced	______	Mirzaie et al. (2020)
	*Coix lachrymal-jobi*	______	______	Diuretic, anti-rheumatic, antispasmodic, anti-inflammatory, antidiarrheal, anthelmintic, antipyretic, antispasmodic, diuretic, hypoglycemic, anti-cancer, and tonic properties (Patel et al. 2017)	______	______	Plant growth study	Li (2003)
Passifloraceae	*Passiflora alata*	*Claroideoglomus etunicatum*, *Rhizophagus intraradices*	Total phenols content	Treatment of several diseases, such as insomnia, anxiety, and hysteria (Simao et al. 2018)	Shoots	______	Dry mass of shoot and leaf number were greater	Riter et al. (2014)
		*Rhizophagus clarus* and *Glomus spurcum*	______			Significant increase	Higher plant height	Riter et al. (2014)
Rutaceae	*Phellodendron amurense*	*Funneliformis mosseae*, *Claroideoglomusetunicatum*, *Diversispora epigaea,* and *Glomus diaphanum*	Berberine, jatrorrhizine, palmatine	Treatment of jaundice, dysentery, hypertension, inflammation, and liver-related diseases (Kuete 2014)	Barks	Significant increase	______	Fan et al. (2006)
	*Phellodendron chinense*	______	Berberine	Treating dysentery, detoxicating, and curing furuncles	______	______	______	Zhou and Fan (2007)
	*Citrus aurantium*	______	______	Possess antiseptic, antioxidant, antispasmodic, aromatic, astringent, carminative, digestive, sedative, stimulant, stomachic and tonic properties Treatment of gastrointestinal disorders, insomnia, headaches, cardiovascular diseases, and cancer (Suryawanshi 2011)		______	Plant growth and root antioxidative enzymes study	Wu et al. (2010)
Solanaceae	*Datura stramonium*	*Funneliformis mosseae* and *Glomus epigaeum*	Hyoscine and hyoscyamine	Treatment of stomach and intestinal pain from worm infestation, toothache, and fever from inflammation (Soni et al. 2012)	Seeds And Fruits	Significant Increase	______	Wei and Wang (1989)
	*Solanum viarum*	*Glomus aggregatum* and bacteria *Bacillus coagulans* and *Trichoderma harzianum*	Flavonoids	Used for cancer, patients with Addison’s disease and rheumatic arthritis treatment	Seedlings	Increased	______	Hemashenpagam and Selvaraj (2011)
	*Withania somnifera*	*Rhizophagus irregularis*	Withaferin-A	Treatment of cancer	Root	Significantly increased	______	Johny et al. (2021)
Taxaceae	*Taxus chinensis*	______	______	Anticancer effect (Jian et al. 2016)	______	______	AMF infection and colonization study	Ren et al. (2008)
Violaceae	*Viola tricolor*	*Rhizophagus irregularis*	Caffeic acid concentration	Treatment of various skin disorders and upper respiratory problems	Aerial part	Significant increase	No impact on root mass and negative impact on shoot biomass	Zubek et al. (2015)
Zingiberaceae	*Curcuma longa*	*Glomus*, *Gigaspora*, and *Acaulospora* sp.	Curcumin	A natural antioxidant with antitumor activity, an inhibitor of arachidonic acid metabolism, and a good antiinflammatory agent	Rhizomes	Increased	______	Dutta and Neog (2016)
		*Gigaspora margarita*	Curcumin		______	No impact on curcumin content (field)	No impact on plant growth parameters, biomass production, nutrient uptake	Yamawaki et al. (2013)
		*Gigaspora margarita*	Curcumin		______	Concentration of curcumin increased (greenhouse)	Higher biomass production and nutrient uptake	Yamawaki et al. (2013)

^a^The column “AMF” shows the current names, not the one at the time of publication

^b^There are no studies or available data found online

A direct relationship has been highlighted between the biomass of AMF-colonized plants and the concentration of secondary metabolites for several medicinal plants, such as *Chlorophytum borivilianum*, *Dioscorea* spp., *Gymnema sylvestre*, *Glycyrrhiza uralensis*, *Libidibia ferrea*, *Ocimum basilicum*, *Satureja macrostema*, and *Salvia miltiorrhiza* (Dave et al. 2011; Lu et al. 2015; Zimare et al. 2013; Chen et al. 2017; Silvia et al. 2014; Zolfaghari et al. 2013; Carreón-Abud et al. 2015; Yang et al. 2017). Conversely, in *Cynara cardunculus* colonized by *R. intraradices* and *F. mosseae*, a significant increase in yield was noticed, but the concentrations of phenolics decreased (Colonna et al. 2016). Other studies conducted with *Hypericum perforatum* inoculated with *R. intraradices* or a mixture of *Funneliformis constrictum*,* Funneliformis geosporum*, *F. mosseae*, and *R. intraradices* reported no increase in shoot biomass, while in *Valeriana officinalis* inoculated with *R. intraradices* or a mixture of six AMF species (*F. mosseae*, *R. intraradices*, *Glomus cladoideum*, *Rhizoglomus microaggregatum*, *Funneliformis caledonium*, and *C. etunicatum*) a negative effect on rhizome and root biomass was noticed (Zubek et al. 2012; Nell et al. 2010). However, an increased concentration of active compounds (e.g., hypericin and pseudohypericin and sesquiterpenic acids, respectively) was noticed for both plants (Zubek et al. 2012; Nell et al. 2010).

Another beneficial aspect of AMF is their capability to improve plant nutrient uptake (Bowles et al. 2016), influencing directly or indirectly the concentration of secondary metabolites (Yamawaki et al. 2013). For instance, *F. mosseae* improved shoot and root biomass, root system architecture, and flavonoid accumulation in *Glycyrrhiza uralensis* growing under P-deficient nutrient conditions (Chen et al. 2017).

A number of studies also have reported enhanced survival and increased growth of micropropagated medicinal plants at the transfer stage from in vitro to ex vivo conditions (Rai 2001). For instance, with *F. mosseae*, the survival rate of micropropagated *Spilanthes acmella* and *Glycyrrhiza glabra* plantlets was 100%, and plant growth and development were improved under glasshouse and greenhouse conditions (Yadav et al. 2012, 2013) while in the absence of AMF, the survival rate was only 60–70%. Similarly, height and fresh weight of shoots, roots, and seeds of *Scutelleria integrifolia* seedlings inoculated with *C. etunicatum* were significantly increased in pots following micropropagation (Joshee et al. 2007).

These studies clearly evidenced the potential of using AMF inoculants for improving the yield of raw materials (e.g., roots, shoots) of medicinal plants, thus potentially increasing the quantity of active compounds.

Different groups of secondary metabolites whose production was enhanced by AMF inoculation are detailed below.

### Alkaloids

Alkaloids are nitrogen-containing organic compounds produced by plants constitutively or in response to pests, diseases, or other external stimuli (Jan et al. 2021). They are found in different organs of important medicinal plants (Table 1) and are characterized by a diverse array of pharmacological properties including analgesia, local anesthesia, cardiac stimulation, respiratory stimulation and relaxation, vasoconstriction, muscle relaxation, antineoplastic, and hypertensive and hypotensive properties (Hussein and El-Anssary 2018).

Since Wei and Wang (1989) first observed that AMF symbiosis can increase the total content of hyoscyamine and scopolamine in *Datura stramonium*, numerous studies have reported a positive role of AMF in the accumulation of alkaloids. For example, a positive correlation was found between AMF colonization (a mixture of *R. intraradices* and *G. margarita*) of *Castanospermum australe* tree and the castanospermine content (which was reported to inhibit the HIV virus) of leaves (Abu-Zeyad et al. 1999). The contents of some commonly used “heat-clearing” herb compounds, such as berberine, jatrorrhizine, and palmatine, were increased in seedlings of *Phellodendron amurense* inoculated with AMF (Fan et al. 2006). Other active compounds were increased in the presence of AMF: trigonelline in roots and leaves of *Prosopis laevigata* colonized by *Gigaspora rosea* under in vitro conditions; colchicine in tubers of *Gloriosa superba* colonized by *F. mosseae* growing under glasshouse conditions, and scopolamine in leaves of *Eclipta prostrata* colonized by a mixture of *C. etunicatum*, *Claroideoglomus claroideum*, *F. mosseae*, *F. geosporum*, *R. irregularis*, and *Rhizoglomus microaggregatum* growing in climate chamber conditions (Rojas-Andrade et al. 2003; Pandey et al. 2014; Vo et al. 2019).

## Terpenoids

The largest and most diverse group of secondary metabolites are terpenoids, which are primary constituents of essential oils (Cox-Georgian et al. 2019). Essential oils are volatile lipophilic mixtures of secondary metabolites, consisting mostly of monoterpenes, sesquiterpenes, and phenylpropanoids, which often are used as flavors and fragrances, as antimicrobials and antioxidants, and as medicines (Deans and Waterman 1993).

Several studies have reported an AMF impact on the production of essential oils by medicinal and aromatic plants (Table 1). For instance, the production of these compounds was increased in *Corianderum sativum*, *Trachyspermum ammi*, *Atractylodes lancea*, *Inula ensifolia*, *Artemisia umbelliformis*, *Plectranthus amboinicus*, *Satureja macrostema*, *Salvia officinalis*, *Origanum vulgare* and *Origanum onites*, *Thymus daenensis*, *Thymus vulgaris*, and *Foeniculum vulgare* colonized by AMF (Rydlová et al. 2016; Kapoor et al. 2002; Liang et al. 2018; Zubek et al. 2010; Binet et al. 2011; Merlin et al. 2020; Carreón-Abud et al. 2015; Sete da Cruz et al. 2019; Karagiannidis et al. 2012; Arpanahi et al. 2020; Machiani et al. 2021; Kapoor et al. 2004). The content of artemisinin, an important sesquiterpene lactone compound found in *Artemisia annua* and well known for its effects on malaria and more recently on cancer (Krishna et al. 2008), was increased in leaves of plants colonized by *F. mosseae* or a combination of *Glomus macrocarpum* and *R. fasciculatus* or *Diversispora epigaea* and *R. irregularis* grown in pots or under field conditions (Huang et al. 2011; Chaudhary et al. 2008; Domokos et al. 2018). The forskolin content, a diterpene extensively used to treat heart diseases, glaucoma, asthma, and certain types of cancers (Kavitha et al. 2010), was significantly increased in roots of *Coleus forskohlii* inoculated with *Glomus bagyarajii* growing under greenhouse conditions (Sailo and Bagyaraj 2005). Similarly, Singh et al. (2013) reported an increased content of forskolin in tubers of *Coleus forskohlii* associated with *R. fasciculatus* growing under organic field conditions.

Researchers also have studied the impact of AMF symbiosis on medicinal plants derived from tissue cultures. An example is the increased content of the essential oil carvacrol, a phenolic monoterpenoid with antimicrobial, antioxidant, and anticancer activities (Sharifi-Rad et al. 2018) in micropropagated *Origanum vulgare* subsp. *hirtum* after association with the AMF *Septoglomus viscosum* (Morone Fortunato and Avato 2008).

### Phenolics

Phenolics represent a wide group of compounds, sharing one or more phenol groups (Hussein and El-Anssary 2018), among which are flavonoids, curcuminoids, coumarins, tannins, stilbenes, lignans, phenolic acids, and quinones (Cosme et al. 2020).

Arbuscular mycorrhizal fungi have been shown to increase the content of phenols in medicinal plants (Table 1). For instance, the production of formononetin (an antimicrobial, antioxidant, antilipidemic, antidiabetic, antitumor, and neuroprotective compound) (Vishnuvathan et al. 2016), was increased in *Medicago sativa* grown in the presence of *R. intraradices* (Volpin et al. 1994). The production of curcumin (an anti-inflammatory, antioxidant, anticancer, antiseptic, antiplasmodial, astringent, digestive, diuretic compound) was increased by circa 26% in *Curcuma longa* colonized by AMF species belonging to the genera *Glomus/Rhizophagus*, *Gigaspora*, and *Acaulospora* sp., under greenhouse conditions (Dutta and Neog 2016). The concentration of total tannins, used to treat tonsillitis, pharyngitis, hemorrhoids, and skin eruptions (Britannica 2021), was increased by 40% in the fruits of *Libidibia ferrea* inoculated with *Acaulospora longula* under field conditions (Santos et al. 2020). Additionally, the concentrations of cichoric acid in *Echinacea purpurea* colonized by *R. intraradices* (Araim et al. 2009) and p-hydroxybenzoic acid and rutin in *Viola tricolor* colonized by *R. irregularis* (Zubek et al. 2015), and the total content of flavonoids in *Libidibia ferrea* colonized by *Gigaspora albida* and gallic acid in *Valeriana jatamansi* colonized by a consortium of three different isolates of *R. intraradices* spp. (Silvia et al. 2014; Jugran et al. 2015) were increased by the AMF symbiosis*.*

## Saponins

Saponins are characterized by a polycyclic aglycone moiety with either a steroid (steroidal saponins) or triterpenoid (triterpenoidal saponins) attached to a carbohydrate unit (a monosaccharide or oligosaccharide chain) (Hussein and El-Anssary 2018). Among these compounds, a few have demonstrated pharmacological properties, such as antitumor, sedative, expectorant, analgesic, and anti-inflammatory (Hussein and El-Anssary 2018). Arbuscular mycorrhizal fungi were reported to enhance the production of saponins in medicinal plants (Table 1). For instance, the content of glycyrrhizic acid, a triterpenoid saponin used to alleviate bronchitis, gastritis, and jaundice (Pastorino et al. 2018), was increased by 0.38–1.07-fold and by 1.34–1.43-fold after 4 and 30 months, respectively, in *Glycyrrhiza glabra* (liquorice) plants colonized by *F. mosseae* and *D. epigaea* alone or in combination, grown in sand under greenhouse conditions (Liu et al. 2007). Similarly, Johny et al. (2021) reported an increase of glycyrrhizic acid concentration in *Glycyrrhiza glabra* inoculated with *C. etunicatum* under greenhouse conditions*.*

## Other chemical compounds

Hypericin and pseudohypericin are naphthodianthrones (anthraquinone derivatives) mainly extracted from *Hypericum* species (Ayan and Cirak 2008). They have many pharmaceutical properties, such as sedatives, antiseptics, and antispasmodics (Baytop 1999). Zubek et al. (2012) reported an increased content of hypericin and pseudohypericin in *Hypericum perforatum* colonized by *R. intraradices* alone or by a mixture of *F. constrictum*, *F. geosporum*, *F. mosseae*, and *R. intraradices*, under greenhouse conditions.

Withaferin-A, a steroidal lactone, traditionally used in ayurvedic medicine (Mirjalili et al. 2009), has a wide range of pharmacological activities including cardioprotective, anti-inflammatory, immuno-modulatory, anti-angiogenesis, anti-metastasis, and anti-carcinogenic properties. Johny et al. (2021) reported that association between the medicinal plant *Withania somnifera* and *R. irregularis* increased the concentration of withaferin-A as compared to non-inoculated plants under greenhouse conditions.

It should be noted, however, that AMF showed a neutral or decreased effect on the production of certain secondary metabolites. For example, Nell et al. (2010) found that *F. mosseae* has no effect on the total concentrations of phenolic and rosmarinic acid in the roots of *Salvia officinalis*; and Geneva et al. (2010) showed that *R. intraradices* decreased total phenol and flavonoid contents in the leaves of *Salvia officinalis*. Similarly, Zubek et al. (2010) reported significant differences in the effectiveness of different AMF species tested in *Inula ensifolia*. An increased production of thymol derivatives was found in plant roots inoculated with *Rhizophagus clarus*, while a decreased production of these metabolites was reported in roots inoculated with *R. intraradices* under greenhouse conditions (Zubek et al. 2010). Moreover, changes in secondary metabolite composition have been observed in medicinal plants inoculated with AMF. For instance, Geneva et al. (2010) observed a modified composition of essential oils and promotion of the relative quantities of bornylacetate, 1,8-cineole, α-thujones, and β-thujones in *Salvia officinalis* associated with *R. intraradices.* Similarly, *Artemisia umbelliformis* inoculated with an alpine microbial community containing *Planticonsortium tenue* (formerly *Glomus tenue*), *R. intraradices*, *G. claroideum/etunicatum*, and a new *Acaulospora* species showed a significant increase of E-ocimene concomitant with a decrease of E-2-decenal and (E, E)-2–4-decadienal (Binet et al. 2011). Therefore, the selection of the most effective AMF strains for improving the accumulation of desirable active compounds needs to be taken into account.

## Effect of AMF on biomass and production of secondary metabolites in medicinal plants under biotic and abiotic stress conditions

Drought, salinity, heavy metals, pests, and diseases can impact plant growth, reducing their biomass (Hashem et al. 2014; Alwhibi et al. 2017) and consequently affecting the production of secondary metabolites. Arbuscular mycorrhizal fungi can increase the tolerance/resistance of plants against those abiotic and biotic stresses, potentially influencing secondary metabolites production (Hashem et al. 2018).

Several studies have shown that AMF symbiosis can improve the growth and secondary metabolite production of medicinal plants under water deficit conditions. For example, a recent study by Machiani et al. (2021) showed that inoculation with *F. mosseae* significantly improved biomass and essential oil content (mainly thymol, p-cymene and γ-terpinene) of *Thymus vulgaris* plants grown in a 2-year field experiment in intercropping with soybean under water deficit conditions. Similarly, Mirzaie et al. (2020) reported that inoculation with *F. mosseae* significantly increased geranial and β-pinene (both belong to oxygenated monoterpenes essential oils) yields of *Cymbopogon citratus* grown in a greenhouse pot experiment under moderate water stress conditions (50% field capacity).

Salt stress stimulates the accumulation of phenolic compounds in plants as a general defense mechanism to stress (Parvaiz and Satyawati 2008). Intriguingly, this abiotic stress is a principal elicitor influencing synthesis of compounds in many herbs (e.g., cinnamic, gallic, and rosmarinic acids in *Thymus vulgaris*; glycyrrhizin in *Glycyrrhiza glabra*; quinic, gallic, and protocatechuic acids in *Polygonum equisetiforme*) (Bistgani et al. 2019; Behdad et al. 2020; Boughalleb et al. 2020). A recent study by Amanifar and Toghranegar (2020) reported that moderate salt stress stimulated higher production of valerenic acid in *Valeriana officinalis* than a situation without salt stress. Interestingly, this increase was significant when the plants were colonized by *F. mosseae*. Duc et al. (2021) found that a mixture of six AMF species (*C. etunicatum*, *C. claroideum*, *F. mosseae*, *F. geosporum*, *Rhizoglomus microaggregatum*, and *R. intraradices*) increased the tolerance of *Eclipta prostrata* under moderate salt stress in a pot experiment under controlled conditions, inducing major changes in its polyphenol profile.

Minerals, such as cadmium (Cd) and zinc (Zn), also were reported to impact secondary metabolite production in medicinal plants colonized by AMF. For instance, Hashem et al. (2016) observed that an AMF mixture comprising *C. etunicatum*, *F. mosseae*, and *R. intraradices* enhanced the chlorophyll and protein content and considerably reduced lipid peroxidation in *Cassia italica* plants under Cd stress in a pot experiment. Moreover, AMF inoculation caused a further increase in proline and phenol content ensuring improved plant growth under stress conditions.

Arbuscular mycorrhizal fungi symbiosis improved the disease tolerance of medicinal plants through the mediation of secondary metabolites. For instance, Jaiti et al. (2007) reported that a complex of native AMF species increased the tolerance of *Phoenix dactylifera* (a plant characterized by high nutritional and therapeutic value of its fruits (Al-Alawi et al. 2017)) against bayoud disease (the most damaging vascular disease of date palm caused by *Fusarium oxysporum* f. sp. *albedinis*) by increasing the enzymatic activities of peroxidases and polyphenoloxidases, which are associated with an increase of phenolic compounds in the cell wall.

## Mechanisms by which AMF symbiosis promotes secondary metabolism in medicinal plants

It is often considered that the increased concentrations of various secondary metabolite groups (e.g., flavonoids, phenolics) in AMF-colonized plants are a result of the elicitation of several defense response pathways as reviewed by Zeng et al. (2013). For instance, terpenoids in the carotenoid pathway, flavonoids, phenolic compounds, and some alkaloids (such as hyoscyamine and scopolamine) in the phenylpropanoid pathway are often increased in AMF-colonized plants (Kaur and Suseela 2020). These pathways play different roles in the plant-AMF symbiosis, such as signaling, stress tolerance, nutrient uptake, and resistance against biotic and abiotic stresses. However, it is still not totally clear how AMF trigger changes in the concentrations of phytochemicals in plant tissues (Toussaint et al. 2007).

**Fig. 1:**
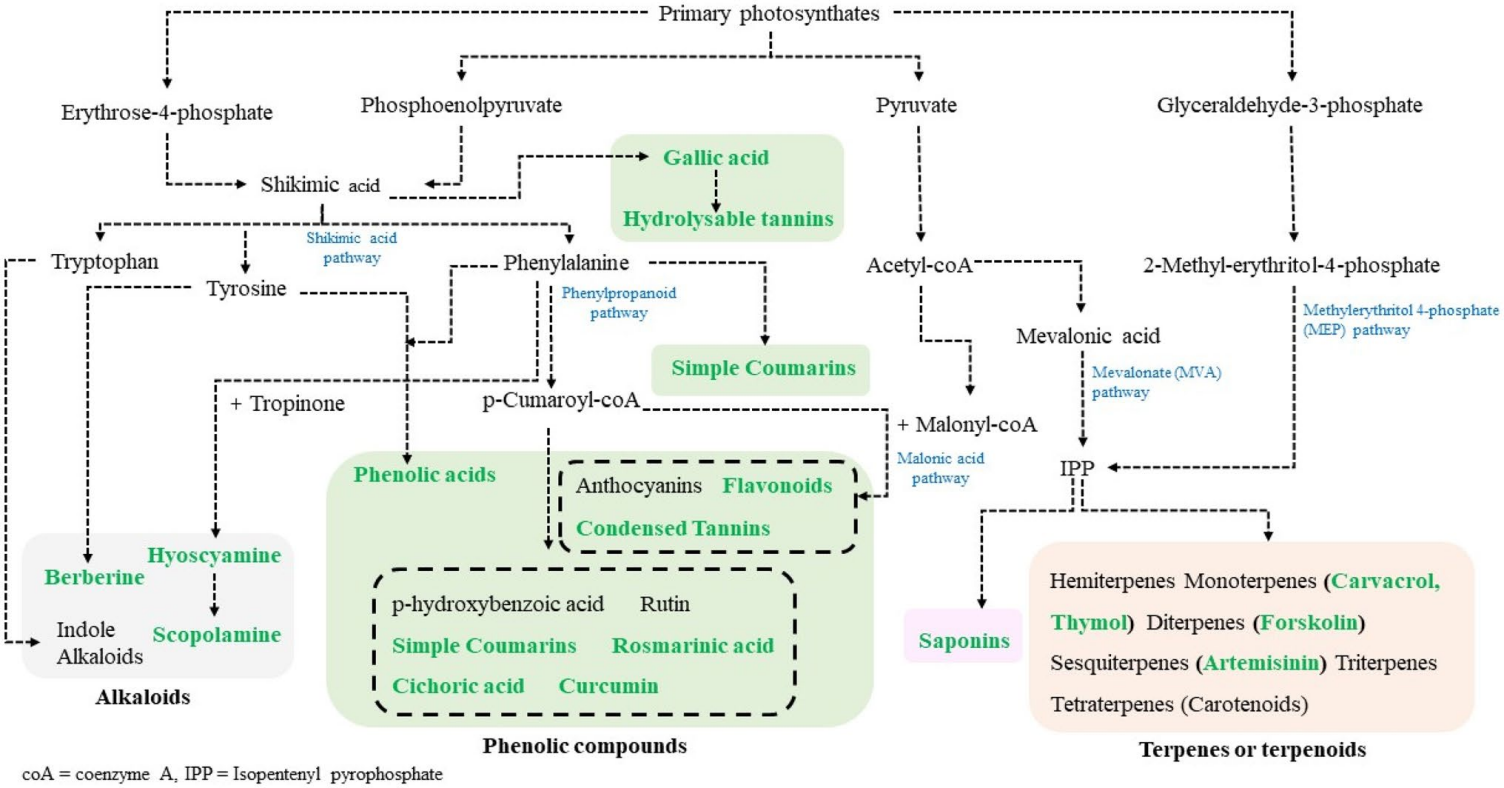
Main pathways of secondary plant metabolism resulting in the production of alkaloids, phenolics, saponins, and terpenes (in gray, green, pink, and brown shaded portions, respectively) mentioned in this review. Examples of upregulated compounds or classes of compounds in medicinal plants associated with AMF are highlighted with green type. This figure is modified from Dos Santos et al. (2021)

Many studies have focused on the mechanisms by which AMF modulate the production of terpenoids, phenolic compounds, and alkaloids in plants. Terpenoids are synthesized from isoprene units in the methyleritrophosphate (MEP) and the mevalonic acid (MVA) pathways (Zhi et al. 2007). Phenolic compounds (e.g., phenols, flavonoids, protanthocyanidins, tannins) are synthesized in the shikimic acid pathway where phenylpropanoids are formed and in the malonic acid pathway (Oksana et al. 2012). Most of the alkaloids are synthesized from various biological precursors (most amino acids) such as tyrosine and tryptophane in the shikimic acid pathway (Facchini 2001) (Fig. 1).

**Fig. 2:**
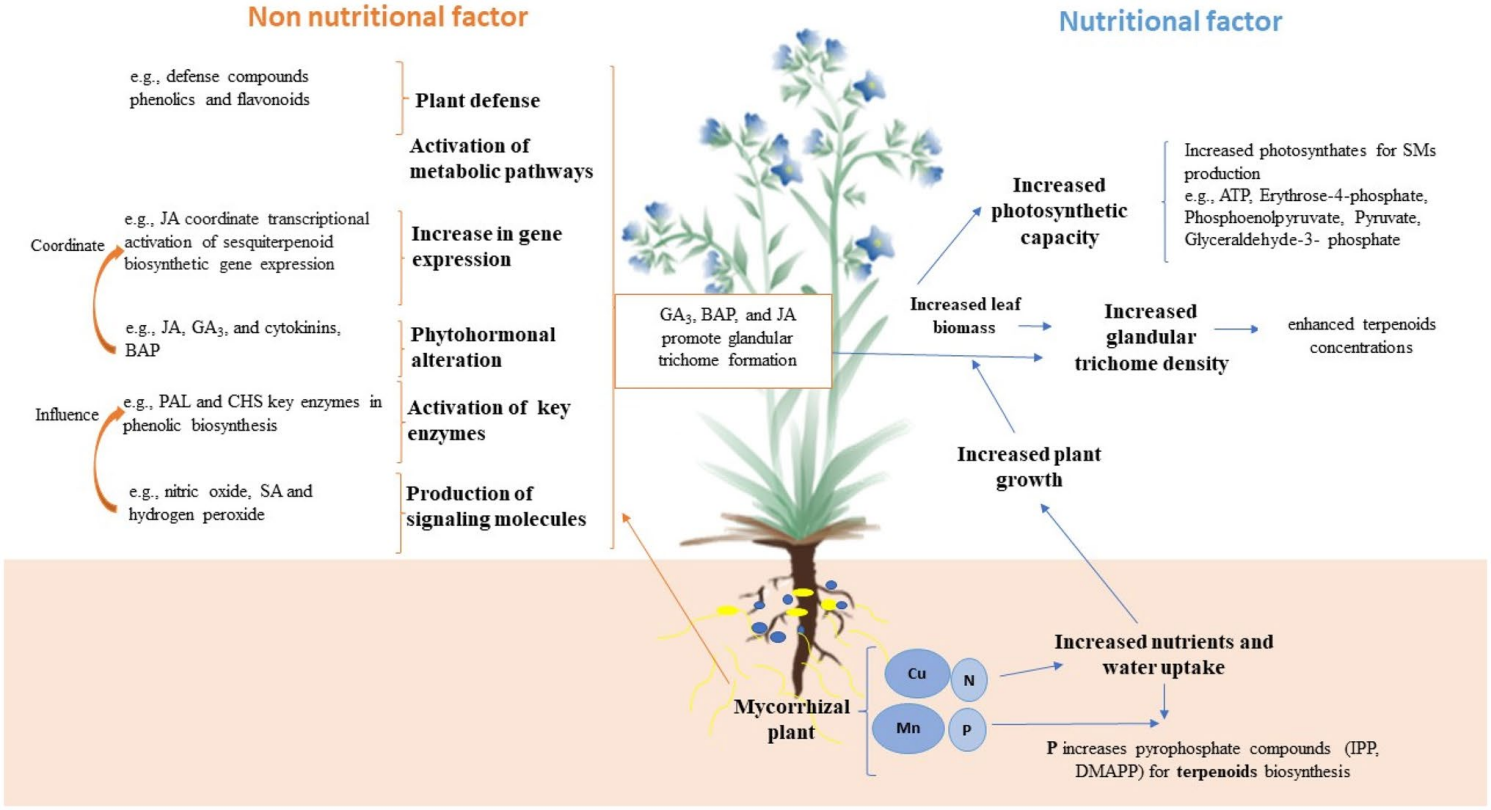
Non-nutritional and nutritional factors influencing the production of secondary metabolites (i.e., terpenoids, phenolics, and flavonoids) in AMF-colonized plants. Non-nutritional factors (leftside in orange): AMF colonization results in the activation of plant defense mechanisms with the production of phenolics and flavonoids. Change in phytohormone levels, such as jasmonic acid (JA), gibberellic acid (GA_3_), and 6-benzylaminopurine (BAP), increases the number and size of glandular trichomes and leads to transcriptional activation of sesquiterpenoid biosynthetic gene expression. AMF induce the production of signaling molecules, such as nitric oxide, salicylic acid (SA), and hydrogen peroxide, which influence the activation of key enzymes such as l-phenylalanine ammonia lyase (PAL) and chalcone synthase (CHS), for the biosynthesis of phenolic compounds. Nutritional factors (rightside in blue): AMF colonization increases plant nutrients and water uptake leading to increased plant growth and leaf biomass. This results in enhanced plant photosynthetic capacity and increased production of photosynthates which are precursors of different secondary metabolites. Increased leaf biomass leads to an increased density of glandular trichomes in which terpenoids are synthesized and stored. This figure is adapted with permission from Springer Nature Customer Service Centre GmbHS: Springer Nature, Phytochemistry Reviews. Insight into the mechanisms of enhanced production of valuable terpenoids by arbuscular mycorrhiza (Kapoor et al. 2017). We thank Evangelia Tsiokanou (National and Kapodistrian University of Athens, Greece) for graciously providing the picture of the plant used in this figure

Several common nutritional and non-nutritional factors have been proposed to explain the increased production of secondary metabolites in AMF-colonized plants (Kapoor et al. 2017; Sharma et al. 2017; Dos Santos et al. 2021) (Fig. 2).

Regarding nutritional factors, the increase was first attributed to the enhanced uptake of nutrients by AMF-colonized plants (Lima et al. 2015; Oliveira et al. 2015; Riter et al. 2014). For example, the role of phosphorus in the synthesis of terpenoids precursors via the MVA (acetyl-CoA, ATP, and NADPH) as well as the MEP (glyceraldehyde phosphate and pyruvate) pathways is widely recognized (Kapoor et al. 2017). Phosphorus enhances terpenoid biosynthesis by increasing the concentration of pyrophosphate compounds, such as isopentenyl pyrophosphate (IPP) and dimethylallyl pyrophosphate (DMAPP) (Kapoor et al. 2002, 2004; Zubek et al. 2010), which contain high-energy phosphate bonds. However, Khaosaad et al. (2006) found that the concentration of essential oils significantly increased in two *Origanum* sp. genotypes colonized by *F. mosseae*, while the levels of essential oils in plants treated with P did not change. This suggests that the increased concentration of essential oils in AMF-colonized *Origanum* sp. plants may directly depend on the association with the fungus. In another study by Zubek et al. (2012), AMF colonization improved hypericin and pseudohypericin concentrations in *Hypericum perforatum*, probably because of an improved plant P and/or N nutrition in presence of the fungi. The increased growth through improved nutrients and water uptake of AMF-colonized plants also explains the enhanced production of these compounds in plants. It is well known that the AMF symbiosis increases shoot biomass, shoot length, and number of nodes in *Ocimum basilicum* (Gupta et al. 2002; Khaosaad et al. 2008; Rasouli-Sadaghiani et al. 2010; Copetta et al. 2006). Elevated leaf biomass results in increased photosynthetic capacity (Dave et al. 2011; Zubek et al. 2010), thus increasing the production of total photosynthates (e.g., ATP, carbon substrate, glyceraldehyde-3-phosphate, pyruvate, phosphoenolpyruvate, or erythrose-4-phosphate) required for terpenoids, phenolics, and alkaloid biosynthesis (Cao et al. 2008; Hofmeyer et al. 2010; Niinemets et al. 2002).

Regarding non-nutritional factors, alterations in the levels of phytohormones in AMF-colonized plants may reflect their enhanced production (Mandal et al. 2013, 2015a; Zubek et al. 2012). Indeed, it has been shown that the AMF symbiosis changes the concentrations of phytohormones, such as jasmonic acid (JA), gibberellic acid (GA_3_), and cytokinins (Allen et al. 1980, 1982; Hause et al. 2002; Shaul-Keinan et al. 2002) in plants. Moreover, it has been reported that phytohormones play a role in the secondary metabolism of plants (An et al. 2011; Maes and Goossens 2010; Maes et al. 2008). For instance, JA has been reported to coordinate transcriptional activation of sesquiterpenoid biosynthetic gene expression in *Artemisia annua* (Maes et al. 2011). Furthermore, the phytohormonal alterations of GA_3_, BAP (6-benzylaminopurine), and JA have been reported to promote the formation of glandular trichomes (Maes et al. 2011) which is positively correlated with an enhanced concentration of terpenoids in plant leaves. Glandular trichomes are the epidermal secretory structures in which terpenoids are synthesized and stored in plants (Covello et al. 2007). The enhanced concentration of terpenoids (essential oils) and increased glandular trichome density has been observed in a number of plants (e.g., *Mentha x piperita*, *Phaseolus lunatus*, and *Lavendula angustifolia*) (Ringer et al. 2005; Bartram et al. 2006; Behnam et al. 2006). Thus, an increase in trichome density upon mycorrhization often has been linked with an enhanced concentration of terpenoids (Copetta et al. 2006; Kapoor et al. 2007; Morone-Fortunato and Avato 2008). The modification of these secondary metabolite concentrations in AMF-plants also may be due to signaling mechanisms between host plants and the fungi (Larose et al. 2002; Rojas-Andrade et al. 2003; Xie et al. 2018). For example, Zhang et al. (2013) have reported that *F. mosseae* associated with *Trifolium repens* promoted changes in the concentration of signaling molecules, such as nitric oxide, salicylic acid (SA), and hydrogen peroxide, which influence the activation of key enzymes in phenolics biosynthesis (e.g., l-phenylalanine ammonia lyase (PAL), and chalcone synthase (CHS)). Moreover, AMF may increase the expression of genes encoding enzymes leading to the biosynthesis of these compounds in mycorrhizal plants (Andrade et al. 2013; Battini et al. 2016; Mandal et al. 2015a, b; Xie et al. 2018). For example, induction of terpene synthase (TPS) family genes TPS31, TPS32, and TPS33 has been observed in AMF-colonized tomato plants and probably can explain the changes in their terpenoid profile (Zouari et al. 2014). Mandal et al. (2015a) reported the increase of artemisinin in leaves of *Artemisia annua* inoculated with *R. intraradices*. This result was correlated with a higher expression of key biosynthesis genes (such as an allene oxidase synthase gene encoding one of the key enzymes for JA production) via enhanced JA levels. In addition, AMF may enhance the biosynthesis of these compounds either by increasing the production of precursors through the induction of metabolic biosynthetic pathways (Lohse et al. 2005; Zimare et al. 2013; Dos Santos et al. 2021) and/or by induction of key synthase enzymes (Mandal et al. 2013; Shrivastava et al. 2015; Sharma et al. 2017; Dos Santos et al. 2021). For example, mycorrhizal colonization (*R. intraradices*) has been found to elevate the transcript levels of two of the pivotal enzymes of the MEP pathway, 1-deoxy-D-xylulose 5-phosphate synthase (DXS), and 1-deoxy-D-xylulose 5-phosphate reductoisomerase (DXR) in wheat roots (Walter et al. 2000). DXS is an enzyme that catalyzes the initial step of the MEP pathway, where many isoprenoids are biosynthesized, and DXR is an enzyme that is immediately downstream from DXS in the MEP pathway (Walter et al. 2000). In another study by Walter et al. (2002), DXS2 transcript levels were strongly stimulated in *Medicago truncatula* roots upon colonization by AMF (a mixture of *F. mosseae* and *R. intraradices*), and were correlated with the accumulation of carotenoids and apocarotenoids. Finally, alterations in these secondary metabolites’ production also can result from plant defense responses to AMF colonization (Mechri et al. 2015; Zubek et al. 2012, 2015; Torres et al. 2015).

Various studies have reported increased production of alkanin/shikonin and their derivatives (A/S) in cell cultures of Boragenaceous plants (e.g., *Lithospermum erythrorhizon*, *Alkanna tinctoria*, and *Arnebia euchroma*) after applying exogenous jasmonate (Gaisser and Heide 1996; Urbanek et al. 1996; Bychkova et al. 1993). Alkanin/shikonin are naphtoquinone compounds with a broad spectrum of biological activities, such as wound healing, anti-inflammatory, and anticancer (Kheiri et al. 2017; Kourounakis et al. 2002; Andújar et al. 2013). Interestingly, AMF colonization of various other plants, such as *Hordeum vulgare*, *Cucumis sativus*, *Medicago truncatula*, and *Glycine max*, has resulted in the increase of endogenous levels of jasmonates within roots (Hause et al. 2002; Vierheilig and Piché 2002; Stumpe et al. 2005; Meixner et al. 2005). Jasmonic acid and its derivatives, commonly termed jasmonates, are hormonal regulators involved in plant responses to abiotic and biotic stresses as well as in plant development (Creelman and Mullet 1997; Wasternack 2007). The level of endogenous jasmonate was shown to increase after wounding or pathogen attack. There is no direct study on the effects of AMF on A/S production in these Boragenaceous medicinal plants. However, these findings suggest that AMF could be a potential factor enhancing A/S production in mycorrhizal Boraginaceae plants through the regulation of jasmonate.

## Cultivation techniques for secondary metabolite production in mycorrhiza-associated medicinal plants

Plant secondary metabolites are often extracted from individuals grown in nature. For instance, around 95% of the medicinal plants used in the Indian herbal industry today are collected from the wild (Lakshman 2016). However, the quantity and quality of secondary metabolites from plants grown in nature are erratic, often influenced by abiotic and biotic factors, such as extreme temperatures, drought, alkalinity, salinity, and plant pathogens, impacting the metabolic pathways responsible for the accumulation of bioactive substances (Dayani and Sabzalian 2017; Giurgiu et al. 2017; Ramakrishna and Ravishankar 2011). Furthermore, overharvesting of medicinal plant species in nature could place them at a high risk of extinction (Roberson 2008). Finally, growing medicinal plants under field conditions may be time consuming, especially for woody plants (e.g., *Taxus brevifolia* and *Lithospermum erythrorhizon*) and slow-growing perennial plants (e.g., *Panax ginseng*), which can take several years to reach the desired metabolites production (Malik et al. 2011; Chandran et al. 2020; Yazaki 2017; Murthy et al. 2014). Therefore, there is a need for alternative production systems.

Production of medicinal herbs in controlled environments provides opportunities for improving the quality, purity, consistency, bioactivity, and biomass production of the raw material (Hayden 2006). In order to secure the commercial production of secondary metabolites, several cultivation techniques have been developed, potentially compatible with AMF application.

## Substrate-based cultivation systems

### Greenhouse cultivation

Greenhouses are widely used for crop production all-year round. Environmental parameters (e.g., temperature, humidity) are controlled, providing optimal growth conditions to the target crop or plant, favoring development, and thus safeguarding the yield and consistent production of high-quality bioactive compounds (Panwar et al. 2003).

Many medicinal plant species, such as *Echinacea angustifolia*, *Echinacea purpurea*, *Ocimum basilicum*, *Withania somnifera*, and *Psoralea croylifolia*, have been grown under greenhouse conditions (Zheng et al. 2006; Panwar et al. 2003). Similarly, many, such as *Artemisia annua*, *Curcuma longa*, *Coleus forskohlii*, *Glycyrrhiza glabra*, and *Gloriosa superba*, have been associated with AMF under greenhouse conditions with high production of bioactive compounds reported (Huang et al. 2011; Dutta and Neog 2016; Sailo and Bagyaraj 2005; Liu et al. 2007; Pandey et al. 2014). Therefore, growing medicinal plants in association with AMF under greenhouse conditions could represent a suitable method for improving the quality and production of bioactive compounds at large scale.

## Substrate-free cultivation systems

### Aeroponics

**Fig. 3:**
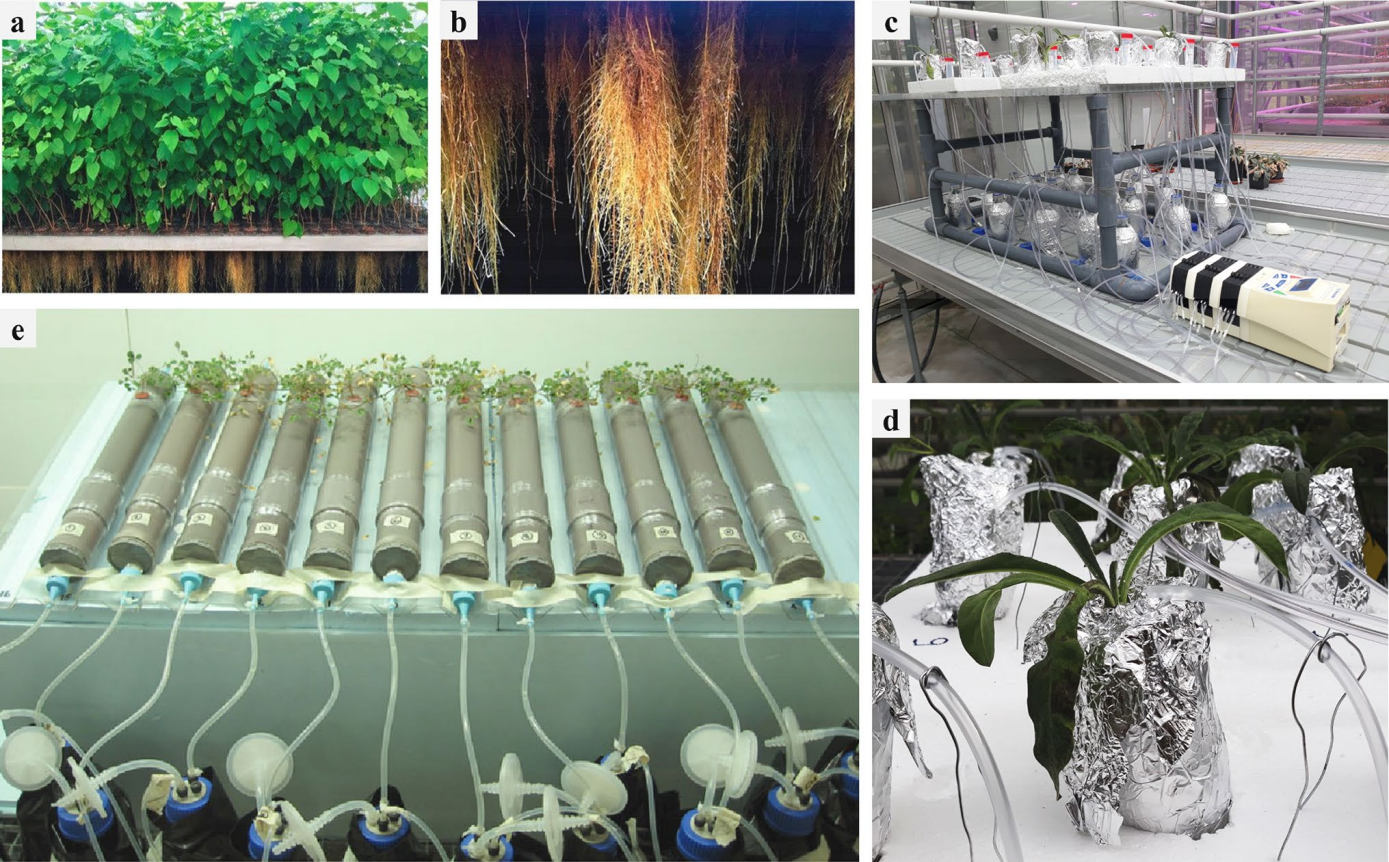
(**a**) *Morus alba* trees cultivated in aeroponic conditions and (**b**) close-up view of *Morus alba* roots grown aeroponically (Chajra et al. 2020). (**c**) *Anchusa officinalis* associated with *Rhizophagus irregularis* MUCL 41,833 growing in a semi-hydroponic cultivation system and (**d**) close-up view of a plant (UCLouvain, greenhouse). (**e**) Plant-based bioreactor system for the mass production of AMF as described in Declerck et al. (2009) (WO/2009/ 090,220)

In the aeroponics cultivation system, the roots of plants are hung inside a sealed container in darkness and exposed to a water nutrient-rich spray through atomizers (Lakhiar et al. 2018) (Fig. 3a). This technique has been developed for the cultivation of many different plants, such as horticultural crops (e.g., *Lactuca sativa*, *Cucumis sativus*, and *Solanum lycopersicum*) (Movahedi and Rostami 2020), medicinal herbs (e.g., *Anemopsis californica*, *Crocus sativus*, and *Valeriana officinalis*) (Hayden 2006; Souret and Weathers 2000; Tabatabaei 2008), and medicinal crops (e.g., *Arctium lappa* and *Zingiber officinale*) used to extract secondary metabolites from their roots (Hayden et al. 2004a, b). It has been reported that *Ocimum* *basilicum* grown under aeroponic conditions had a higher yield, comparable phenolic and flavonoid contents, and antioxidant properties compared to plants grown in a solid substrate (Chandra et al. 2014). Similarly, *Cichorium intybus*, *Withania coagulans*, and *Echinacea* sp. grown in an aeroponic system had higher yields compared to the same plants grown in soil (Movahedi and Rostami 2020). This system was efficient for the production of bioactive molecules from roots of medicinal crops, such as chlorogenic acid in *A. lappa* and β-sitosterol in *Cannabis sativa* (Hayden 2006; Ferrini et al. 2021). For various medicinal plants, root apices constitute the main sites where active substances are produced and stored (Watson et al. 2015). However, these active substances are almost impossible to harvest through conventional farming methods. By using the Plant Milking Technology (Plant milking®) (https://www.plantadvanced.com/home) for *Morus alba* (an emblematic tree of traditional Chinese medicine, rich in alkaloids and flavonoids), Chajra et al. (2020) obtained an extract enriched in prenylated flavonoids that was 18-fold higher than commercial root extracts (Fig. 3a, b).

Interestingly, aeroponic cultivation systems also have been developed and used for the production of AMF inoculum in which roots (and AMF) were bathed in a nutrient solution mist (Zobel et al. 1976; Hung and Sylvia 1988). For the production of AMF, plants are precolonized prior to their introduction into the system, through preculturing plant seedlings and AMF propagules (both preferably surface-sterilized) in pots containing a substrate (e.g., mixture of sand and perlite). Then the precolonized plants are transferred to the aeroponic container where the roots (and AMF) develop. The container is usually protected from light to prevent the development of algae (Jarstfer and Sylvia 1995). The mist can be applied by various techniques that differ mainly in the size of the fine droplets produced (e.g., atomizing disk, pressurized spray through a microirrigated nozzle, an ultrasonically generated fog of nutrient solution with droplets of 3–10-μm diameter, and ultrasonic nebulizer technology resulting into microdroplets of 1 μm in diameter) (IJdo et al. 2011; Jarstfer and Sylvia 1995; Mohammad et al. 2000). Mohammad et al. (2000) reported a high number of viable AMF propagules obtained in aeroponic culture, and such inoculum was used in a field experiment (Mohammad et al. 2004). Thus, aeroponic systems could potentially be used for growing medicinal plants associated with their AMF partners in order to obtain substantial biomass and production of secondary metabolites.

## Hydroponics

Hydroponic systems include all systems that deliver nutrients in liquid, with or without a solid medium to anchor plant roots (Hayden 2006) (Fig. 3c). Such systems have been applied to several medicinal plants, such as *Echinacea angustifolia*, *Ocimum basilicum*, *Leonurus quinquelobatus*, *Mentha piperita*, *Salvia officinalis*, *Achillea millefolium*, *Bidens tripartite*, *Leonurus sibiricus*, *Linum usitatissimum*, *Hypericum perforatum*, and *Tanacetum parthenium* (Maggini et al. 2012; Mairapetyan et al. 2018; Simeunovic 2002). Thanks to these systems, the biosynthesis of active compounds, such as tropane alkaloids in *Datura innoxia*, total phenols and rosmarinic acid in *Ocimum basilicum*, and oil production in *Valeriana officinalis*, has been obtained (Gontier et al. 2002; Sgherri et al. 2010; Tabatabaei 2008).

Different hydroponic culture systems also exist for the mass production of AMF. They mainly differ in the mode of aeration and application of the nutrient solution (IJdo et al. 2011). For instance, in the static hydroponic culture system, the nutrient solution is not flowing and needs to be aerated via an aeration pump to prevent roots of mycorrhizal plants from suffering oxygen deprivation (IJdo et al. 2011). Via this system, Dugassa et al. (1995) obtained large quantities of mycorrhizal *Linum usitatissimum* plant roots as well as extramatrical mycelium and chlamydospores free of residues from solid substrate components. In another nutrient film technique (NFT) hydroponic system, a thin nutrient solution (i.e., film) flows into inclined channels (also called gulls) where the plant roots and AMF develop (IJdo et al. 2011). This technique has been used to culture AMF since the 1980s with the production of many sporocarps by *F. mosseae* (Elmes and Mosse 1984). Later, IJdo et al. (2011) developed an innovative low-cost in vitro plant-based bioreactor system for the mass production of AMF. In this system, *Medicago truncatula* roots and AMF (*Glomus* sp.) were grown in a sterilized tube connected at both extremities to a reservoir containing sterilized liquid culture medium. This nutrient solution circulates across the mycorrhizal root system, feeding the plant/fungus associates, while the plant shoot develops in open-air conditions inside a controlled growth chamber (Fig. 3e). The hydroponic system also has been developed for studying the effect of the AMF symbiosis (e.g., P uptake) on maize plants (Garcés-Ruiz et al. 2017). Therefore, hydroponic or semi-hydroponic systems could potentially be combined with medicinal plants and AMF in order to obtain increased production of secondary metabolites. In a recent study, Cartabia et al. (2021) showed how the *R. irregularis* modified the primary and secondary metabolism and the root exudates of the medicinal plant *Anchusa officinalis* growing under a semi-hydroponic cultivation system (Fig. 3c). Moreover, permeabilization treatments can be conducted in these cultivation systems, in order to extract the compounds exuded by roots in a non-destructive process that “milks” the same plants several times a year. For example, in the study by Gontier et al. (2002), *Datura innoxia* plants were cultivated in hydroponic conditions (no AMF were involved) and the plant roots subsequently permeabilized with Tween 20. As a result, a high concentration of tropane alkaloids (TA) (e.g., hyoscyamine and scopolamine) was detected in the nutrient solution. Interestingly, all the plants were able to survive after being rinsed and replaced in the hydroponic system. This approach allows the permeabilization of the plant multiple times without loss of viability (Gontier et al. 2002). Moreover, different permeabilization treatments (e.g., doses and duration of Tween 20, addition of TA precursors) can be chosen to release additional bioactive compounds in the nutrient solution (i.e., TA precursors (phenylalanine and ornithine) leading to 10–80 mg/l TA in the nutrient solution) (Gontier et al. 2002). This study, however, did not include association with AMF.

## In vitro production systems

Micropropagation or in vitro propagation is the clonal propagation of plants by tissues, cells, or organs. It involves the aseptic culture of explants of tissues or organs in closed vessels using defined culture media in a controlled environment (Debnath and Arigundam 2020).

## Whole plant in vitro culture

In vitro cultivation of whole plants is widely used for mass propagation, conservation of germplasm, production of bioactive compounds, and genetic improvement of a large number of medicinal plant species (Nalawade and Tsay 2004). For instance, protocols have been developed for the in vitro mass propagation of *Limonium wrightii*, *Adenophora triphylla*, *Gentiana davidii*, *Anoectochilus formosanus*, *Scrophularia yoshimurae*, *Pinellia ternata*, *Bupleurum falcatum*, *Zingiber zerumbet*, *Dendrobium linawianum*, and *Fritillaria hupehensis* via shoot morphogenesis, for *Angelica sinensis* and *Corydalis yanhusuo* via somatic embryogenesis, and for *Taxus mairei*, *Angelica dahurica*, *Angelica sinensis*, *Dioscorea doryophora*, *Gentiana davidii*, and *Bupleurum falcatum* via cell suspension cultures (Nalawade and Tsay 2004).

**Fig.4:**
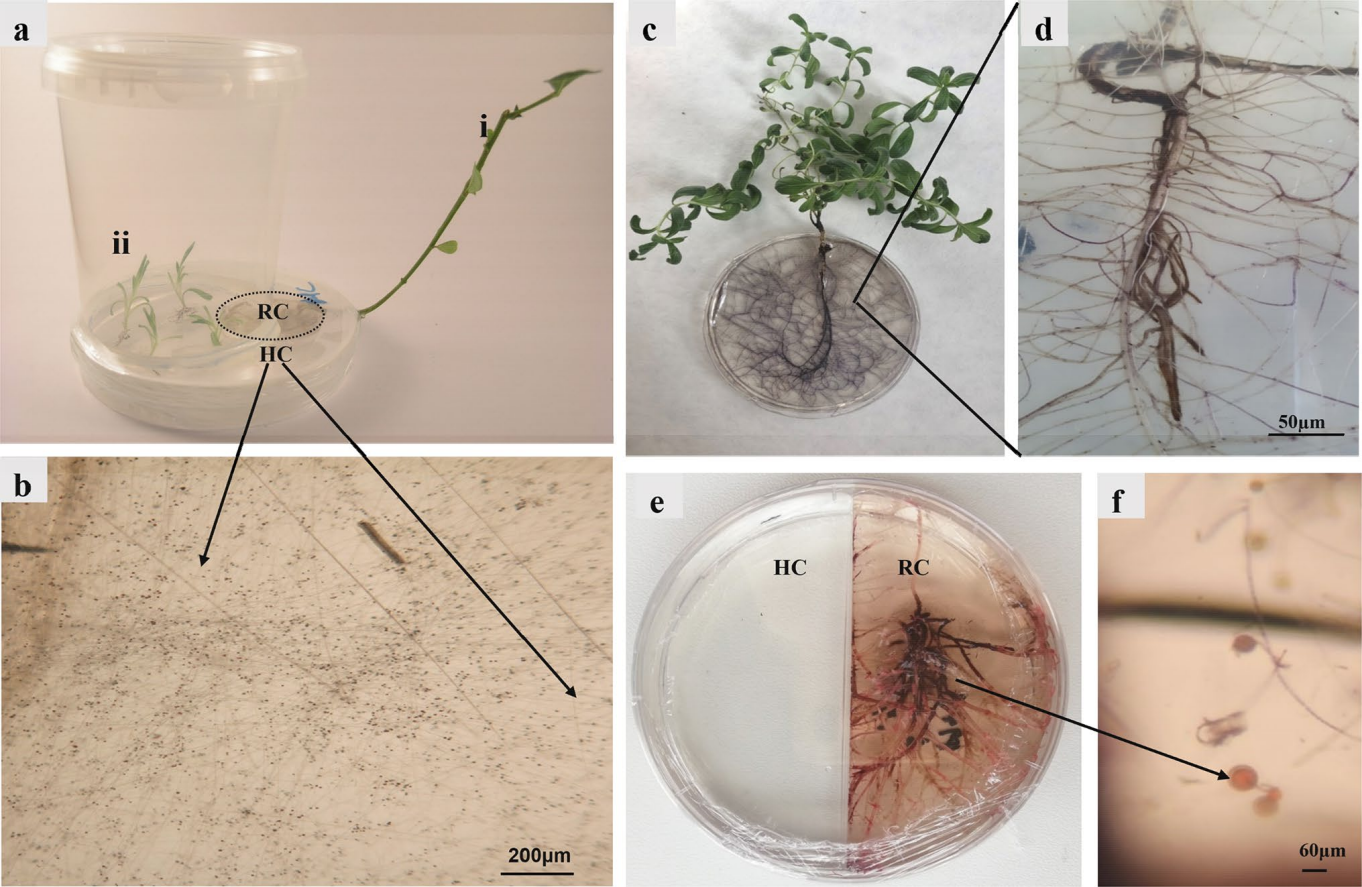
(**a**) A 145-mm mycorrhizal donor plant in vitro culture system. (**i**) The donor plant is *Crotalaria spectabilis* growing in a root compartment (RC) in close association with the arbuscular mycorrhizal fungus *Rhizophagus irregularis* MUCL 41833 and (**ii**) the receiver plants are *Alkanna tinctoria* growing under a lid in a hyphal compartment (HC) in which only a profuse, active extraradical mycelium network proliferates; (**b**) close-up view of extensive development of extraradical mycelium and spores in the HC; (**c**) a 90-mm half-closed arbuscular mycorrhizal plant in vitro culture system allowing the growth of the roots of *Lithospermum erythrorhizon* in close association with *R. irregularis* MUCL 41833; (**d**) close-up view of the reddish roots due to shikonin production; (**e**) a 90-mm root organ culture in vitro system allowing the growth of Ri T-DNA transformed *A. tinctoria* hairy root (Rat et al. 2021) in assocation with *R. irregularis* MUCL 41833 in the RC; (**f**) close-up view of the red AMF spores produced in the RC (arrows). We thank Alicia Varela Alonso (Institut für Pflanzenkultur, Germany) for graciously providing the pictures **c** and **d** and Angélique Rat (Ghent University, Belgium) for providing the *Alkanna tinctoria* hairy roots used in this figure. The system (**a**) starts with a donor plant (*Crotalaria spectabilis*) introduced into the RC of a bi-compartmented system (a small Petri dish indicated by a dashed circle (RC) (90 mm diameter)) placed in a large Petri dish (HC) (145 mm diameter). A hole is made in both Petri dishes allowing the shoot to extend outside the system. Approximately 500 spores from an AMF in vitro culture are placed in contact with the roots. The roots and AMF are kept in the dark during the whole growth period, while shoots remain under light. Once the donor plant is well colonized, the extraradical mycelium starts to cross the partition wall separating the RC from the HC, developing profusely in the HC. At that time, one or several receiver micropropagated plants (*Alkanna tinctoria*) are placed in the HC with their roots in contact with the extraradical mycelium. The plants are planted inside the HC under a lid. Briefly, the base of a cylinder (150 mm high, 100 mm diameter) matches a hole made in the lid of the 145-mm Petri dish. The cylinder top is glued to a 100-mm Petri dish lid. The culture dishes containing the *A. tinctoria* plants are sealed and covered, up to the base of the cylinder, by black plastic bags. The systems are incubated in a growth chamber to allow plant and AMF growth (detailed procedures of this system can be found in Lalaymia and Declerck (2020)). For system (**c**), homogenously chopped agar containing AMF propagules from an AMF in vitro culture is inoculated to the newly growing roots of a micropropagated seedling of *Lithospermum erythrorhizon*. After a few days, the new hyphae growing from the spores colonize the roots of *L. erythrorhizon.* In system (**e**), fine root structures of Ri T-DNA transformed *Alkanna tinctoria* hairy roots are cut and placed in the RC part of a bi-compartmental Petri dish. Chopped agar containing AMF propagules is spread on the young parts of the hairy roots. After a few days, new hyphae growing from spores colonize the *A. tinctoria* hairy root, producing new spores and extensive mycelium after several months. All these three techniques should be conducted under a laminar flow hood with sterilized laboratory materials

The association of AMF with whole plants in vitro has been described in several studies (e.g., Dupré de Boulois et al. 2006; Voets et al. 2005; Lalaymia and Declerck 2020). For instance, using the mycorrhizal donor plant in vitro cultivation system, Voets et al. (2009) obtained fast and homogenous mycorrhization of *Medicago truncatula* seedlings by placing the plantlets in an actively growing mycelial network arising from a mycorrhizal donor plant (Fig. 4a). In another system, called the half-closed arbuscular mycorrhizal plant in vitro culture system, roots of micropropagated potato plantlets were associated with actively growing AMF propagules, while the shoots developed in open-air conditions (Voets et al. 2005) (Fig. 4c). Applying both systems, several thousand spores of *R. intraradices* were produced on an extensive extraradical mycelium and abundant root colonization has been obtained. Hence, they could be extended to medicinal plants to enhance secondary metabolite production (Fig. 4).

## Hairy root cultures (HRCs)

Hairy root cultures are cultures raised after the infection of explants/cultures by the gram-negative soil bacterium *Agrobacterium rhizogenes*^2^ (Tepfer and Casse-Delbart 1987). This bacterium leads to the neoplastic growth of roots which are characterized by high growth rates in hormone-free media and genetic stability (Pistelli et al. 2010). Ri T-DNA transformed hairy roots grow faster than adventitious roots, or even conventional cultures in which plants are growing in soil or substrate (Paek et al. 2009; Yu et al. 2005). For instance, *Panax ginseng*, a valuable medicinal plant originating from Asia, has been used as a healing drug and health tonic since ancient times (Tang and Eisenbrand 1992) due to its production of triterpene saponins, collectively called ginsenosides (Dewick 1997; Huang 1999). However, it generally takes 5 to 7 years in the field to attain maturity and to reach the harvesting stage for extraction of bioactive compounds (Murthy et al. 2014). To solve this problem, many different techniques have been explored, such as culture of callus tissues, suspended cells, adventitious roots, and HRCs (Furuya et al. 1973, 1983; Paek et al. 2009; Shi et al. 2021). It has been reported that HRCs grow more rapidly and produce a higher level of ginsenosides than the suspended cells and adventitious root cultures (Inomata et al. 1993; Yoshikawa and Furuya 1987). Moreover, HRCs produce the same phytochemical patterns as the wild-type root organs and accumulate higher levels of certain valuable compounds compared with adventitious roots and native-grown plant roots (Kai et al. 2011; Hao et al. 2020; Miao et al. 2017). For instance, the total tanshinone content reached 15.4 mg/g dry weight (DW) in transgenic *Salvia miltiorrhiza* hairy roots, while only 1.7–9.7 mg/g DW tanshinone was produced in roots of field-grown plants (Kai et al. 2011; Hao et al. 2020). High stability and productivity, high biomass production, and efficient biosynthetic capacity make HRCs valuable biotechnological tools for the production of plant secondary metabolites (Pistelli et al. 2010; Gutierrez-Valdes et al. 2020). Some examples of metabolites produced using HRCs are tropane alkaloids, such as scopolamine and hyoscyamine (Jouhikainen et al. 1999; Häkkinen et al. 2016; Guo et al. 2018; Khezerluo et al. 2018), catharanthine (Hanafy et al. 2016), ginsenosides (Woo et al. 2004; Ha et al. 2016), solanoside (Putalun et al. 2004), and anthraquinones (Perassolo et al. 2017). The studies mentioned here, however, did not involve inoculation with AMF.

In order to upscale production and commercialize secondary metabolites, various conventional bioreactors, broadly classified as liquid phase, gas phase, or hybrid reactors, have been employed for the mass production of HRCs, which permit the growth of interconnected tissues normally unevenly distributed throughout the vessel. For instance, increased production of terpenoid indole alkaloid and artemisinin was obtained with HRCs of *Rauwolfia serpentina* and *Artemisia annua* grown in different reactors (no AMF involved) (Mehrotra et al. 2015; Patra and Srivastava 2014). Interestingly, the in vitro large-scale production of AMF is mostly based on HRCs (Declerck et al. 1996; Declerck 2006) which involve the association of AMF propagules with transformed hairy roots on synthetic mineral media. Arbuscular mycorrhizal fungi species grown in HRCs produce viable and contaminant-free spores (Fortin et al. 2005) (Fig. 4e). From these studies, we can suggest HRCs of medicinal plants associated with AMF in bioreactors for the commercial production of secondary metabolites.

## Conclusions

Arbuscular mycorrhizal fungi may confer several benefits to medicinal plants, such as growth promotion and improved tolerance to stress conditions. Interestingly, AMF also may enhance the accumulation of active substances in those plants. This makes mycorrhizal technology a potential and sustainable tool for improving the growth and secondary metabolite production of medicinal plants. Factors such as light, temperature, humidity, soil fertility, and cultivation techniques also could influence secondary metabolite production by medicinal plants (Szakiel and Pączkowski 2011a, b). It is thus essential to consider these parameters in fine-tuning the conditions for optimal production of plants and associated secondary metabolites. In order to guarantee the quality of the bioactive substances produced by mycorrhizal medicinal plants, different substrate or substrate-free systems were described: aeroponic and hydroponic or semi-hydroponic systems, micropropagated medicinal plants in half-closed arbuscular mycorrhizal-plant or mycorrhizal donor**-**plant in vitro culture systems, and HRCs. These systems may provide adequate environmental conditions to the plants, resulting in improved crop yield and production of bioactive compounds (Nazari Deljou et al. 2014; Dayani and Sabzalian 2017).

Whatever the system considered, high yields of secondary metabolites are dependent on the AMF strain, the plant, and the environmental growth conditions. Arbuscular mycorrhizal fungi are not host specific, but their affinity to a particular host can be preferential (Cesaro et al. 2008). Early studies have documented that different AMF species may induce differences in secondary metabolite production in the same host or genotype (Zeng et al. 2013). For example, *Glomus caledonium* increased rosmarinic acid and caffeic acid production in *Ocimum basilicum*, whereas *F. mosseae* enhanced only caffeic acid production (Toussaint et al. 2007). In a recent study, Frew (2020) showed that inoculation with four commercial AMF species (*C. etunicatum*, *Funneliformis coronatum*, *F. mosseae*, and *R. irregularis*) had stronger effects on cereal crop plant allometric partitioning, foliar nutrient, and phenolic concentrations than inoculation with a single commercial AMF species (*R. irregularis*). Interestingly, the results also showed that the effects of inoculating with these four commercial AMF species were not different from the effects of applying a native AMF inoculant extracted from field soil, suggesting that commercial AMF assemblages may provide little to no additional benefit compared with a resident AMF community (Frew 2020). Thus, a thorough study of AMF species native to medicinal plants should be conducted before choosing the most effective AMF species (native or not; single or combinations of different AMF strains) to inoculate target plant species.

The use of commercial inoculants is an option which should be considered with caution. Indeed, a number of studies have shown that the absence of a regulatory context for the industry of AMF inoculants may have contributed to inoculants of questionable quality. For instance, Salomon et al. (2022) reported that over 80% of tested commercial inoculants contained no viable propagules when screened in sterilized soil. Moreover, when preparing AMF inoculum, adequate phytosanitary controls must be implemented to avoid proliferation of unwanted microbes which may subsequently contaminate plant production. The use of root organs in vitro may provide a solution by avoiding the presence of such contaminants. However, genetically modified plants may represent a drawback for field application and thus for commercial mass production, and the number of species grown in this system remains limited (Ijdo et al. 2011).

Finally, whatever system is used, environmental parameters should be considered seriously. For instance, light intensity is known to strongly impact the development of AMF. Konvalinková and Jansa (2016) reported that a sudden decrease of light availability to an AMF-colonized plant resulted in a rapid decrease of P transfer from the AMF to the plant, and when arbuscular mycorrhizal plants were exposed to long-periods of shading (weeks to months), positive mycorrhizal growth responses often declined. Ballhorn et al. (2016) also reported that under light limited conditions, vegetative and reproductive traits were inhibited in AMF inoculated *Phaseolus lunatus* plants relative to non-colonized plants. Thus, in controlled conditions, light intensity and quality (e.g., blue/red ratio) should be modulated to improve and guarantee the symbiosis between AMF and plants of interest (Konvalinková and Jansa 2016). Adequate timing and harvestable plant parts also are crucial factors to increase the production of secondary metabolites. The best time to harvest (quality peak season/time of day) should be determined according to the quality and quantity of biologically active constituents rather than the total vegetative yield of targeted medicinal plant parts. Taking into consideration all these environmental factors would help optimize plant-AMF associations, increasing biomass and secondary metabolite production.

## Footnotes

1 The species names of AMF shown in this review follow the current nomenclature and not that of the original publications.

2 Now called *Rhizobium rhizogenes.*
